# Specific gene expression profiles and chromosomal abnormalities are associated with infant disseminated neuroblastoma

**DOI:** 10.1186/1471-2407-9-44

**Published:** 2009-02-03

**Authors:** Cinzia Lavarino, Nai-Kong V Cheung, Idoia Garcia, Gema Domenech, Carmen de Torres, Miguel Alaminos, Jose Rios, William L Gerald, Brian Kushner, Mike LaQuaglia, Jaume Mora

**Affiliations:** 1 Developmental tumour biology laboratory, Hospital Sant Joan de Déu, Fundació Sant Joan de Deu, Barcelona, Spain; 2 Department of Pediatrics, Memorial Sloan-Kettering Cancer Center, New York, NY, USA; 3 Department of Pathology, Memorial Sloan-Kettering Cancer Center, New York, NY, USA; 4 Department of Surgery, Memorial Sloan-Kettering Cancer Center, New York, NY, USA; 5 Biostatistics and Epidemiology, Universitat Autònoma, Barcelona, Spain; 6 Department of Histology, University of Granada, Granada, Spain

## Abstract

**Background:**

Neuroblastoma (NB) tumours have the highest incidence of spontaneous remission, especially among the stage 4s NB subgroup affecting infants. Clinical distinction of stage 4s from lethal stage 4 can be difficult, but critical for therapeutic decisions. The aim of this study was to investigate chromosomal alterations and differential gene expression amongst infant disseminated NB subgroups.

**Methods:**

Thirty-five NB tumours from patients diagnosed at < 18 months (25 stage 4 and 10 stage 4s), were evaluated by allelic and gene expression analyses.

**Results:**

All stage 4s patients underwent spontaneous remission, only 48% stage 4 patients survived despite combined modality therapy. Stage 4 tumours were 90% near-diploid/tetraploid, 44% MYCN amplified, 77% had 1p LOH (50% 1p36), 23% 11q and/or 14q LOH (27%) and 47% had 17q gain. Stage 4s were 90% near-triploid, none MYCN amplified and LOH was restricted to 11q. Initial comparison analyses between stage 4s and 4 < 12 months tumours revealed distinct gene expression profiles. A significant portion of genes mapped to chromosome 1 (*P *< 0.0001), 90% with higher expression in stage 4s, and chromosome 11 (*P *= 0.0054), 91% with higher expression in stage 4. Less definite expression profiles were observed between stage 4s and 4 < 18m, yet, association with chromosomes 1 (*P *< 0.0001) and 11 (*P *= 0.005) was maintained. Distinct gene expression profiles but no significant association with specific chromosomal region localization was observed between stage 4s and stage 4 < 18 months without MYCN amplification.

**Conclusion:**

Specific chromosomal aberrations are associated with distinct gene expression profiles which characterize spontaneously regressing or aggressive infant NB, providing the biological basis for the distinct clinical behaviour.

## Background

In 1971, a special and rare subgroup of metastatic NB affecting very young infants and characterized by a unique pattern of dissemination and a high incidence of spontaneous regression was described [1]. This subgroup, designated stage 4s (s for special), has been recognized as a distinct clinical entity in all subsequent classifications of NB [2].

Historically, stage 4s is defined as a small primary tumour (usually resectable and not crossing the midline) in children <1 year of age, with no bony metastasis and minimal marrow involvement [2]. Conversely, infant stage 4 NB typically includes extensive marrow involvement, bony metastasis and a large primary tumour. Accuracy of staging is critically important since chemotherapy is standard of care for stage 4, while minimal or no therapy is most appropriate for stage 4s patients. However, clinical distinction between 4s and 4 can be difficult, especially among patients with 1) distant but no bony metastases, 2) distant MIBG positivity, 3) <10% marrow disease because of the heterogeneity of tumour infiltration and 4) large primary tumours that cross the midline. Furthermore, based on recent studies the long-accepted 1-year age cut-off at diagnosis for infant stage 4 NB is now being redefined to all patients up to18 months of age [3,4]. Objective, biological markers are clearly needed to distinguish spontaneously regressing tumours from those with stage 4 NB.

Various combinations of prognostic markers have been used with some success for this stage distinction, including ploidy, histology, and genetics [5-8]. Yet there remain cases that were clinically compatible with 4s stage but not fully compliant with the expected marker profile [9-12]. As a result, uniform therapeutic strategies remain difficult to implement, and some stage 4s cases may receive unnecessary cytotoxic therapy. It is also possible that some infant stage 4s NB may masquerade as clinical stage 4 tumours, and if left alone, could undergo spontaneous remission. The identification of biological markers that allow a precise distinction of these NB subgroups will help accurate classification.

Here, we evaluate by allelic and gene expression analyses infants with stage 4 and 4s NB, uniformly staged and treated at Memorial Sloan Kettering Cancer Centre (MSKCC) from 1987 to 2000. Our results show chromosomal abnormalities and specific gene expression profiles associated with each infant NB subgroup. These results provide new insights in the biology of infant NB, useful to enable the identification of markers that can be reliably used to distinguish spontaneously regressing stage 4s from infant stage 4 NB.

## Methods

### Patients and samples

A total of 35 primary infant NB (25 stage 4 < 18 months and 10 stage 4s) obtained at diagnosis from patients treated at MSKCC from 1987 to 2000 were selected for allelic and gene expression analyses (see Additional file 1). Patients were evaluated by CT, ^99m^Tc bone scan, ^131^I MIBG scan, bilateral bone marrow aspirates and biopsies, and measurement of urinary and serum tumour markers. Since 1987, a conservative approach was adopted at MSKCC for stage 4s NB, based on surgery rather than upfront chemotherapy or radiation therapy [13]. Infant stage 4 NB tumours were treated with chemotherapy according to N5, N6 or N7 protocols [14]. All specimens were examined by the same pathologist (W.G.) for tumour cell content and INPC classification [15]; only tumours with > 70% neuroblastic cells were included in the study. The specimens were obtained and processed uniformly. This study was approved by the MSKCC Institutional Review Board and informed consent was obtained before collection of all samples.

### Allelic analysis

Genomic DNA was extracted following standard procedures. Polymorphic microsatellite loci were identified in the Genome Data Base. Map locations were obtained from previous reports [17] and NIH Genemaps. From the patients analyzed, immunocytology screened tumour free peripheral blood cells or frozen bone marrow, were used as normal tissue counterparts [16]. Analysis was performed using fluorescently-labelled primers (Research Genetics, Birmingham, AL) following described procedures [18,19].

### *MYCN *and DNA content analyses

*MYCN *gene copy number was determined as previously reported [20]. *MYCN *was considered amplified when the ratio of the target gene copy number and single-copy reference gene was >5-fold by Southern blot. The modal DNA content was determined by flow cytometry [21]. DNA index (DI) was expressed as the ratio of tumour DNA content/standard DNA fluorescence; near-diploid DI = 0.90–1.20; near-triploid DI = 1.21–1.75; near-tetraploid DI = 1.76–2.20.

### Gene expression profiling

Gene expression analysis was carried out as previously reported [22,23]. Total RNA was extracted from snap-frozen tissues and labelled by linear amplification with biotinylated nucleotides and detected with fluorescent labeled avidin. The quality of labelled cRNA target was assessed by hybridization to Affymetrix test II arrays. Gene expression was carried out using Affymetrix Human Genome GeneChip U95Av2 array using instruments and protocols recommended by the manufacturer. Microarray data and sample annotations have been deposited in caArray, Array Data Management System, National Cancer Institute (https://array.nci.nih.gov/caarray/home.action; Experiment ID: lavar-00111).

### Gene expression data analysis

Genes with high variability within samples were selected, for screening purposes, by pair-wise comparison analyses, controlling the multiple tests by the False Discovery Rate (FDR) approach [24] equivalent to a q-values calculations with λ = 0 [25], and their PPV (Predictive Positive Value) defined as 1-FDR, and with no type-I error adjustment (Raw method) for classic p-values calculations. The cut-off Family-wise error applied to select significant genes by means of the T-test for independent data, a univariate screening supervised procedure, was <0.01. The aim of this screening procedure was to provide multiple sets of genes for hierarchical clustering analyses. The hierarchical clustering was performed with normalised data using Ward's algorithm and the square of Euclidean distance as metrics; analyses were performed for the differentially expressed genes selected in the previous step, taking into account the relationship between gene expressions. Fisher's exact test and 95% bilateral confidence interval using Wilson method were used to evaluate the proportion with which chromosomes were represented in the selected gene sets in comparison to chromosome representation within the Affymetrix GeneChip U95Av2. Statistical analyses were performed using SAS 9.1 and JMP 5.1 (SAS Institute Inc) for Windows and CIA 2.1.1.

### Gene Ontology annotation categories

Gene Ontology (GO) annotation categories were analyzed usingExplore Gene Ontology (*e*GOn v2.0) in *Gene Tools *web service http://www.genetools.no. Only categories in the "biological process" section were used. Overrepresented GO annotations were determined statistically by Fisher's exact test; *P*-value cut-off was set at 0.05. *P*-values from Fisher's exact test were adjusted for multiple testing (False Discovery Rate) using the Benjamini-Hochberg step-up procedure; cut-off was set at 0.05.

### Quantitative Real-time Polymerase Chain Reaction

Quantification of transcript levels was performed for 6 of the differentially expressed genes (see Additional file 2). Concomitant *MYCN *gene copy number analysis was performed. DNA and RNA were isolated by standard procedures. Q-PCR reactions and quantification, using the ΔΔC_T _method for relative quantitation, were performed on an ABI Prism 7000 Sequence Detection System using TaqMan^® ^Assay-on-Demand Gene Expression products (Applied Biosystems, USA). Transcript levels were measured relative to those of 3 normal tissue samples (adrenal gland, lymph node and bone marrow) and normalized to the expression of TATA-binding protein (*TBP*). *MYCN *gene copy number quantification was performed as reported previously [26]. Gene copy number was calculated relative to placental DNA using the B-Cell maturation factor (BCMA) as reference gene. All experiments included no template controls and were performed in duplicate and repeated twice independently.

## Results

### Patient and tumour characteristics

The clinicobiological characteristics of the patients are shown in (see Additional file 1). Median age at diagnosis was 12.96 and 2 months for stages 4 and 4s, respectively. Metastases to distant sites other than bone marrow were common among stage 4 patients (25/25), with preponderance to bone (21/25), less to liver (8/25), and uncommon to skin (2/25) or CNS (2/25). All evaluable stage 4s patients had liver involvement, 40% bone marrow, 30% skin and none CNS. Stage 4 tumours were predominantly unfavorable histology cases, whereas all stage 4s tumours were histologically favorable. DNA index was available for 31 cases (21 stage 4 and 10 stage 4s). A majority of stage 4 tumours (90%) were in the near-diploid (16/21) or near-tetraploid (3/21) range. In contrast, 9 of the 10 stage 4s tumours showed hyperdiploid (near-triploid) clones, and case #1 was the only diploid stage 4s NB. *MYCN *copy number was amplified in 11/25 stage 4 cases, but no stage 4s was *MYCN *amplified. Seven of the 10 stage 4s patients had clinical high-risk features at presentation. In particular, patient #3 had extensive tumour burden at diagnosis: unresectable thoracoabdominal primary tumour, massive liver involvement, peritoneal implants, pleural nodules and metastases to bone marrow (<10%), skin and testes. Overtime, 6 stage 4s patients had progressive disease. At the time of progression, biopsies were carried out in two cases (# 6 and 8) and resections of the primary tumour in three patients (# 1, 2 and 7). All four 4s patients with bone marrow disease underwent spontaneous regression without cytotoxic therapy. The time to last positive bone marrow (as measured by histology) ranged from 3 to 9 months from diagnosis, when the age of the patients was from 7 to 15 months. All 10 stage 4s patients had liver involvement that also regressed spontaneously. The time to last positive liver CT scan/ultrasound ranged from 6 to 38 months from diagnosis (age from 13 to 38 months). All 10 stage 4s patients are alive, disease-free, median follow-up of 170 months. Eight of the 10 stage 4s patients never received cytotoxic therapy. Patients # 1 and 9 received a single cycle of chemotherapy (cyclophosphamide-doxorubicin and carboplatin-etoposide, respectively) and patient #9 450 cGy to the liver, because of respiratory failure requiring intubation. In contrast, 12 of the 25 stage 4 patients (48%) are alive, disease-free (median follow-up of 150 months for stage 4 patients currently alive, median follow-up of 92 months for all stage 4 patients).

### Allelic and *MYCN *analyses

Adequate samples were available for 31 (9 stage 4s, 14 stage 4 *MYCN *nonamplified (NA) and 8 stage 4 *MYCN *amplified (A)) of the 35 cases selected for allelic analyses. Specific patterns of allelic loss (LOH) were associated with stage 4s and stage 4 tumours (see Additional file 1). Chromosome arm 1p loss was restricted to stage 4 patients and represented the most common region of LOH, 17/22. The highest incidence of loss was on distal 1p36, encompassing the previously described shortest region of overlap for LOH [17], and was detected for 11/22 stage 4 cases, of which 6/11 *MYCN *A and 5/11 *MYCN *NA tumours. Chromosome 11q LOH was observed in 5/22 of stage 4 patients, none with *MYCN *amplification and/or 1p36 LOH, similar to previous reports [27]. Amongst *MYCN *nonamplified stage 4 tumours, 1p22 and 1p34-p31 LOH was observed in association with 11q and/or 14q loss. Four out of 8 stage 4s tumours also exhibited 11q LOH. Chromosome 14q LOH of at least one locus was identified in 6/22 stage 4 tumours, a majority *MYCN *NA, while chromosome arm 14q was preserved in all 9 stage 4s tumours. Chromosome 17q allelic analysis was performed in 23 of the 35 cases. Gain of chromosome 17q was identified in 8/17 stage 4 NB, 4/10 *MYCN *NA and 4/7 *MYCN *A cases (see Additional file 1).

Examples of LOH, allele preservation and allelic imbalance in an aneuploid tumour were shown in prior publications [28].

### Differential gene expression analysis

Pair-wise comparison analysis was performed initially for 20 primary infant NB tumours diagnosed at < 12 months; owing to the small number of cases, stage 4 NB were analyzed together without distinction of *MYCN *gene status (8 stage 4s and 12 infant stage 4, including 5 *MYCN *amplified cases).

**Figure 1:**
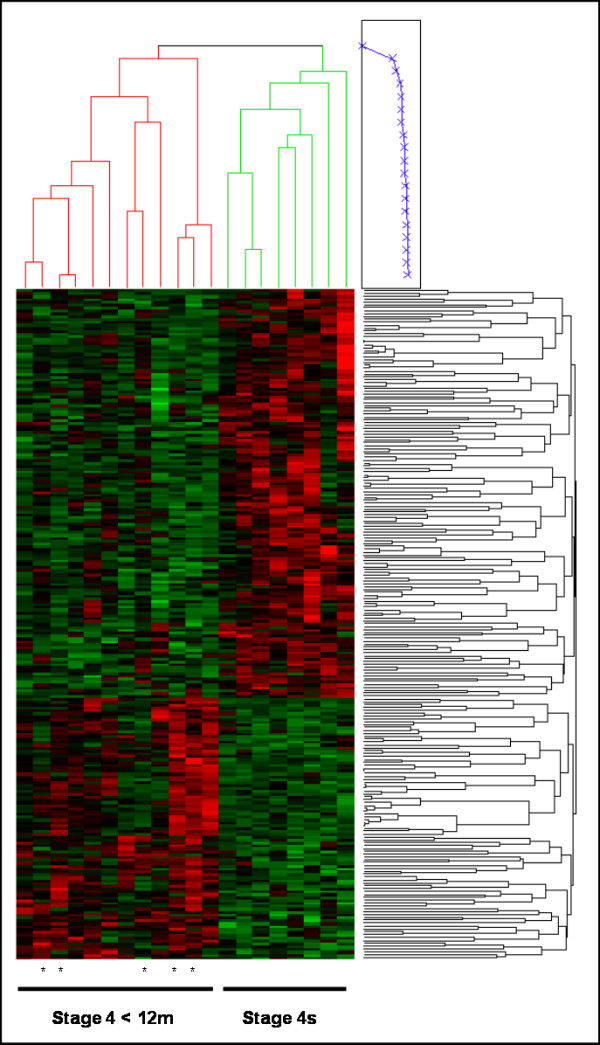
**Hierarchical clustering and heatmap representation of expression profiles associated with 231 differentially expressed genes identified by comparison analysis between stage 4s and stage 4 NB tumours < 12 months**. *: stage 4 MYCN amplified tumours.

The analysis revealed 231 genes with statistical significant different expression levels (RAW *P *< 0.01; FDR between 0.22 – 0.53) (see Additional file 3 Table A). Hierarchical clustering and heatmap representation of distinct expression profiles associated with each NB subgroup is shown in Figure 1. A statistically significant portion of the differentially expressed genes mapped to chromosome 1, (16.7% *P *< 0.0001); chromosomal regions 1p36-p34, 1p21-p13 and 1q21.2-q42; a majority (90%) showed higher expression in stage 4s NB. Chromosome 11, appreciably overrepresented with a substantial set of differentially expressed genes (10.7% *P *= 0.0054), exhibited elevated expression levels nearly exclusively (91.3%) in stage 4 NB. Chromosome 17 differentially expressed genes (6.4% *P *= 0.5635) showed region specificity related to each NB subgroup; chromosome 17p13-17q21 genes (60%), showed increased expression in stage 4s NB, whereas, genes located to 17q21-q25 (40%) showed higher expression in stage 4 NB.

Gene Ontology functional classification of genes displaying higher expression in stage 4 tumours showed enrichment for genes related to cellular metabolism, RNA metabolism, protein, macromolecular and nucleic acid biosynthesis, whereas, stage 4s included genes involved in cell communication and signal transduction.

Gene expression profile comparisons between stage 4s and stage 4 NB diagnosed at < 18 months (19 cases), identified 220 statistically significant differentially expressed genes (RAW analysis, *P *< 0.01; FDR 0.046 – 0.54) (see Additional file 3 Table B). Heatmap representation of differentially expressed genes revealed a less definite expression pattern within stage 4 tumours (see Additional file 4). Specific chromosomal gene location remained statistically significant, chromosomes 1 (17.8% *P *< 0.0001) and 11 (9.8% *P *< 0.01), and association with the NB subgroups was maintained. Genes located on chromosome 2 were also overrepresented (8.9%) with high expression levels identified exclusively in stage 4s NB. As for stage 4 <12m, GO classification revealed for stage 4 < 18m enrichment for genes related with biosynthetic processes, while stage 4s analysis disclosed genes involved in cellular developmental process and immune response.

Despite the small set of tumours, differential gene expression between stage 4s and stage 4 < 18 months *MYCN *not amplified tumours (9 cases) was also investigated. Distinct expression profiles were generated by 107 differentially expressed genes (RAW *P *< 0.01; FDR value equal to 0.896) (see Additional file 3 Table C and Additional file 5). Again, genes located on chromosomes 1 and 11 were over represented without reaching statistical significance. No statistically significant overrepresentation of GO annotations was found.

Similarities in mRNA expression between the three pair-wise comparisons were assessed in an intersectional Venn diagram analysis of expressed gene probe sets (see Additional file 6). The number of probe sets common between stage 4s *versus *stage 4 < 12m and stage 4s *versus *stage 4 < 18m was higher than with stage 4s *versus *stage 4 <18m MYCN NA (124/233 (53%) and 124/224 (55%) *versus *51/107 (47%) and 48/107 (45%) probe sets, respectively), underlining the influence of MYCN amplification on gene expression profiles. Taken together, 38 probe sets were found common among all three gene lists. Although our study strongly suggests that infant stage 4 and stage 4s NB harbour distinct gene expression profiles, the small cohort of cases may have led to an overestimation of the differentially expressed genes.

### Quantitative Real-time Polymerase Chain Reaction (Q-PCR)

**Figure 2:**
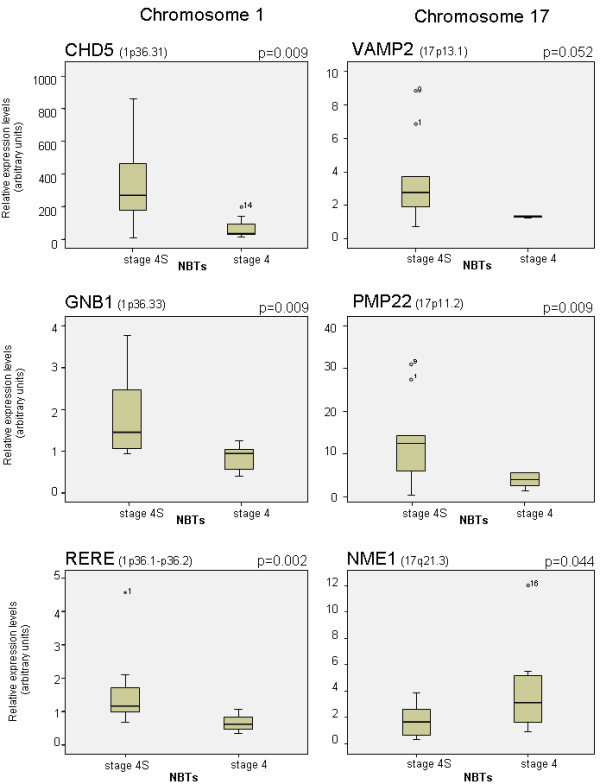
**Quantitative real-time PCR validation of microarray gene expression data**. Comparison of gene expression levels of 6 representative genes located on chromosomes 1 and 17 for 19 infant NB tumours (12 stage 4s and 7 stage 4 < 18m). Results were compared by two-tailed independent-sample *t *test using SPSS v.14.0 for Windows (SPSS, Chicago, IL). Expression data are shown as box plots (SPSS v.14.0).

Quantification of transcript levels of 6 differentially expressed genes located on the chromosomes 1p and 17p13-q21 was performed for 19 infant NB tumours (12 stage 4s and 7 stage 4 < 18m) with available adequate specimens. Considering the reduced number of cases available for this analysis, Q-PCR analysis confirmed microarray differential gene expression for all the analyzed genes (Figure 2).

## Discussion

The dichotomy of spontaneous remission *versus *lethal metastases has branded NB as clinically erratic. The discovery by D'Angio et al. in 1971 [1], that certain infant NB had a special clinical course was a stunning observation providing the basis for reducing cytotoxic therapy for specific subgroups of NB [1]. However, as recently as 1998, patients with 4s NB were treated with chemotherapy because of an uncertainty that these can evolve into malignant stage 4 disease [29]. "High risk" stage 4s tumours, that will nonetheless spontaneously regress, may represent the "tip of the iceberg" among young children between 1 to 2 years of age, diagnosed with non-skeletal metastatic NB. Further insights in the biology of infant NB are clearly important to enable the identification of markers that can be reliably used to distinguish spontaneously regressing stage 4s from infant stage 4 NB.

Tumour markers such as *MYCN *amplification, histology, ploidy, and NTRK1 expression have proven prognostic value in large cohorts of NB patients. The *MYCN *oncogene is generally single-copy in stage 4s tumours but recent studies of infant NB have identified some cases of *MYCN*-amplified tumours otherwise compatible with clinical stage 4s [17,30-32]. Stage 4s tumours consistently have favourable histology; most cases show near-triploid clones and are TrkA positive. Yet, these features also occur in stage 4 disease. In a large multi-institutional study of infants with NB, near-diploid and near-triploid tumours were found among both stage 4 and 4s [33]. Our results suggest that the presence of near-triploid clones in the tumour is a useful indicator of stage 4s biology in infants and may be helpful in accurate classification even for cases that may be considered clinically high risk. Other markers such as serum ferritin [34], E-rosette inhibition factor [34], class I MHC antigen expression [35], telomere length [36] and telomerase activity [37] have been reported to differ for the two NB groups but are of limited clinical utility.

Since the early 1980's, cytogenetic studies have shown consistent losses of chromosome arm 1p in advanced stage NB [38]. Subsequent analyses using a variety of molecular techniques have confirmed common LOH on chromosome 1p36, 11q and 14q in advanced stage NB tumours suggesting the location of genes that play an important role in aggressive disease [38]. Furthermore, allelic analyses have supported the existence of at least two loci on 1p that may be important for NB tumourigenesis [38]. In our study, the notable and consistent absence of any chromosome 1p loss in stage 4s tumours agreed with all previously published reports, and represents a distinct difference between stage 4 and 4s tumours.

In the past few years, high-throughput microarray gene expression analysis has contributed to improve classification of numerous neoplasias, identify sets of genes of prognostic importance, define risk-groups and predict therapy response. Hence, microarray technology has been applied to study many aspects of NB [39]. In this report, we describe distinct gene expression profiles related to spontaneously regressing NB and infant stage 4 NB tumours diagnosed at < 18 months. Given the low incidence of stage 4s neuroblastoma (3% of neuroblastoma) few expression profiling studies, with reduced cohorts, have addressed the issue of differential gene expression in infant disseminated NB [40-43]. Among these, no comparison study considering exclusively spontaneously regressing NB and infant stage 4 NB, including two age cut-off at diagnosis (<12 and <18 months) has been reported. In contrast, reported cohorts of stage 4 NB include all age patients, albeit, infant stage 4 NB have a better prognosis than older stage 4 children [44]. Two of these studies [40,42] failed to identify distinct gene expression patterns characteristic of stage 4s *versus *stage 4 using microarray technology; both studies included among stage 4 NB a small sample set of infants. The Fischer et *al *study [43] identified a set of differentially expressed transcripts (~500 transcripts) generated from 8 cases (5 stage 4s and 3 stage 4 >12 months) using SAGE technology. A subsequent validation by Q-PCR analysis performed on a large group of cases (total of 76 samples, 10 stage 4 < 18 months), confirmed 18 genes differentially expressed between stage 4 and 4s. Among the genes identified, Dystonin (DST) on chromosome 6p, and MAP7 on chromosome 6q were also identified in our list of genes (see Additional file 3).

Chromosomal mapping of the identified differentially expressed genes, demonstrated that subsets of genes within each NB group map to specific chromosomal regions. In particular, stage 4s tumours showed higher expression levels of a significant set of genes localized on chromosome 1p when compared both to infant *MYCN*-amplified as well as nonamplified stage 4 NB tumours. Conversely, a significant portion of genes showing increased expression in stage 4 NB, mapped to chromosome 11. Nonrandom loss and gain of whole chromosomes have been reported to characterize near-triploid NB tumours; in particular, loss of whole chromosome 11 has been reported as a recurrent genetic event in near-triploid tumours along with frequent loss of 11q in near-diploid tumours [45]. This gene expression profile could reflect a potential loss of whole chromosome 11 in a portion of stage 4s tumours. Ongoing aCGH studies in our laboratory are confirming these results (data not shown). Clearly, association of differential gene expression with chromosome 11 was not observed when stage 4s tumours were compared to stage 4 *MYCN *NA tumours, owing to the presence of alterations in this chromosome in both NB subgroups. Recently, amongst unfavorable NB tumours, two major genetic subtypes have been described: advanced stage NB without *MYCN *amplification, with high 11q LOH directly associated with 14q deletion, but inversely correlated with tumours with *MYCN *amplification and 1p36 allelic loss [46,47]. Our results confirm this major genetic subdivision, infant stage 4 *MYCN *NA NB exhibit a lower incidence of 1p36 chromosomal losses, and higher 11q LOH associated with 14q LOH with respect to infant *MYCN *A NB, significantly associated with distal 1p36 allelic loss. Interestingly, stage 4 tumours without *MYCN *amplification may exhibit 1p22 and 1p34-p31 LOH in association with 11q and 14q loss, suggesting that losses in these 1p chromosomal regions are not mutually exclusive as for 1p36 LOH.

Our report identifies a distinct gene expression profile between infant stage 4 and 4s NB and regional chromosomal expression patterns correlating with specific genomic abnormalities for each subgroup of tumours. Correlations between genomic abnormalities and expression profiles have recently been reported in many tumour types including neuroblastoma [48], and are relevant to decipher the intricacies of tumorigenesis. A recent study showed the correlation of copy number and overexpression of WSB1 on 17q11.1, more common in stage 4s and locoregional NB compared to stage 4 tumours [41]. We found that chromosome 17 differentially expressed genes exhibited region specificity related to each NB group; chromosome 17p13-17q21 genes showed higher expression in stage 4s NB, whereas, stage 4 tumours displayed higher expression of genes localized to 17q21-q25, possibly reflecting the partial gain of chromosome arm 17q, the most frequent genetic alteration associated with unfavorable NB tumours. Interestingly, three nucleoside diphosphate (NDP) kinases, two located on 17q23.2, *NME1 *and *NME2*, and *NME4 *on 16p13.3, exhibited increased expression levels in infant stage 4. The incremented expression of *NME1 *and *NME2 *has been previously associated with 17q gain, unfavorable NB and *MYCN *overexpression [49]. Our results thus, support the assumption of a major role of nucleoside diphosphate (NDP) kinases in tumourigenesis of infant unfavorable NB. Overall these results, together with the work of Chen et al., 2006 [41] and Lastowska et al., 2002 [50], suggests the importance of the imbalance between the dosage of genes localized on both sides of chromosome 17q breakpoints in the tumourigenesis of (infant) NB.

## Conclusion

According to our preliminary results, distinct chromosomal aberrations may be reflected in gene expression profiles associated with spontaneously regressing or aggressive infant NB, and thus, with the distinct clinical behaviour. The identification of gene expression profiles associated with subgroups of infant disseminated NB warrants further investigation to identify sets of gene useful for better stratification of infant neuroblastoma. The potential to use this information must be tested in larger, prospective, cooperative trials.

## Abbreviations

NB: neuroblastoma; MIBG: Meta-iodobenzylguanidine; LOH: loss of heterozygosity; MSKCC: Memorial Sloan-Kettering Cancer Centre; CT: computed tomography; INSS: International Neuroblastoma Staging System; INPC: International NB pathology committee; CNS: central nervous system; Q-PCR: Quantitative real-time polymerase chain reaction; FDR: False Discovery Rate; PPV: Predictive Positive Value.

## Competing interests

The authors declare that they have no competing interests.

## Authors' contributions

CL, NKC and JM are responsible for the initial conception and overall hypothesis of this study. CL, IG and JM are responsible for the design of this manuscript, including the original draft and subsequent revisions and design of this manuscript. NKC and WLG were involved with the interpretation of data, draft and revision of this manuscript. CdT assisted with the initial concept and was involved with the draft and revisions of this manuscript; provided guidance for many of the experiments. NKC, BK, MLQ and WLG are responsible for the procurement and cryopreservation of NBT tissue specimens derived from MSKCC. IG, CL and JM were responsible for the procurement and cryopreservation of NBT tissue specimens derived from the Spanish institutions. WLG evaluated tumour specimens for staging classification and tumour content. CL, NKC, WLG, and JM are responsible for patient clinico-biological database management and for microarrays studies. IG and CL are responsible for the quantitative PCR experiments. BK and MLQ were also involved in the drafting and revision of this manuscript. All were also involved in the drafting and revisions for this manuscript. All authors read and approved the final manuscript.

## Pre-publication history

The pre-publication history for this paper can be accessed here:

http://www.biomedcentral.com/1471-2407/9/44/prepub

## Supplementary Material

Additional file 1 **Clinicobiological characteristics of all patients studied.** Dx: specimen at diagnosis; rel: specimen at relapse. Primary Tumour: ABD: abdomen; ADR: adrenal gland; ABD+T: abdominal and mediastinal; RP: retroperitoneal. Metastasis: L: liver; MD-LN: Mediastinal lymph nodes; PL: pleural; T: testicular; PS: paraspinal; BM: bone marrow; B: bone; SKN: skin; Br: Brain. Age: at Diagnosis in months. Tx: therapy; * cases included in gene expression analysis; ** one dose of doxorubicin and cyclophosphamide; *** one cycle of carboplatin/VP-16 and 450 cGy to the liver; N5-7: MSKCC protocols; CCG and POG: prior CCG or POG protocols before arriving at MSKCC. -: no chromosomal alteration,LOH: loss of heterozygosity; +: chromosome arm 17q gain; Status: D: dead; A: alive. F/u: follow-up in months. Click here for file

Additional file 2 **Quantitative real-time polymerase chain reaction analysis.** Genes located on chromosomes 1 and 17 analyzed for quantification of differential expression. Click here for file

Additional file 3 **Gene expression profiling.** List of genes identified to be differentially expressed in infant NB. Genes are displayed according to the tumour INSS stage exhibiting higher expression levels and gene chromosomal localization. Table A. Analysis Stage 4s versus Stage 4 < 12 months. List of 231 differentially expressed genes; Raw P < 0,01; Table B. Analysis of Stage 4s versus Stage 4 < 18 months. List of 220 differentially expressed genes; Raw P < 0,01; Table C. Analysis of Stage 4s versus Stage 4 < 18 months without MYCN amplification. List of 107 differentially expressed genes; Raw P < 0,01. Click here for file

Additional file 4 **Hierarchical clustering and heatmap representation of gene expression profiles.** Comparison analysis between stage 4s and stage 4 NB tumours < 18 months: 220 differentially expressed genes. Click here for file

Additional file 5 **Hierarchical clustering and heatmap representation of gene expression profiles.** Comparison analysis between stage 4s and stage 4 NB tumours < 18 months without MYCN amplification: 107 differentially expressed genes. Click here for file

Additional file 6 **Intersectional Venn diagram analysis of differentially expressed gene probe sets.** Intersectional Venn diagram analysis of differentially expressed gene probe set ID reported in Additional file 3. The number of probe sets common between stage 4s *versus *stage 4 < 12m and stage 4s *versus *stage 4 < 18m was higher than with stage 4s *versus *stage 4 <18m MYCN NA; 124/233 (53%) and 124/224 (55%) *versus *51/107 (47%) and 48/107 (45%) probe sets, respectively. Thirty-eight probe sets were found common among all three gene lists. Click here for file
