# Supplementary material for: Specific gene expression profiles and chromosomal abnormalities are associated with infant disseminated neuroblastoma
Source: BMC Cancer. 2009 Feb 3;9:44. doi: 10.1186/1471-2407-9-44 (PMC2642835; doi:10.1186/1471-2407-9-44)
Supplement: Additional file 1 — Clinicobiological characteristics of all patients studied. Dx: specimen at diagnosis; rel: specimen at relapse. Primary Tumour: ABD: abdomen; ADR: adrenal gland; ABD+T: abdominal and mediastinal; RP: retroperitoneal. Metastasis: L: liver; MD-LN: Mediastinal lymph nodes; PL: pleural; T: testicular; PS: paraspinal; BM: bone marrow; B: bone; SKN: skin; Br: Brain. Age: at Diagnosis in months. Tx: therapy; * cases included in gene expression analysis; ** one dose of doxorubicin and cyclophosphamide; *** one cycle of carboplatin/VP-16 and 450 cGy to the liver; N5-7: MSKCC protocols; CCG and POG: prior CCG or POG protocols before arriving at MSKCC. -: no chromosomal alteration,LOH: loss of heterozygosity; +: chromosome arm 17q gain; Status: D: dead; A: alive. F/u: follow-up in months. [file 1471-2407-9-44-S1.doc]

**Additional File 1**: Clinicobiological characteristics of all patients studied.

|  | **Age (m)** | **Primary** | **Metastasis** | **Tx** | **Ploidy** | **MycN** |  | **1p36** | **1p34-p31** | **1p22** | **11q** | **14q** |  | **Status** | **F/u** |
| --- | --- | --- | --- | --- | --- | --- | --- | --- | --- | --- | --- | --- | --- | --- | --- |
| **Tumour #** |  | **Tumour** |  |  |  |  | **1p** |  |  |  |  |  | **17q G** |  |  |
| **STAGE 4S** |  |  |  |  |  |  |  |  |  |  |  |  |  |  |  |
| 1-Dx* | 0.66 | ADR | L | ** | Diploid | NA | - | - | - | - | LOH | - | - | A | 122 |
| 2-Dx* | 3.72 | ADR | L + BM + MD-LN | NO | Triploid | NA | - | - | - | - | LOH | - | - | A | 211 |
| 3-Dx | 0.20 | CHEST | L+PL+BM+SKN+T | NO | Triploid | NA | - | - | - | - | - | - |  | A | 50 |
| 4-Dx* | 5.95 | ABD | L + BM | NO | Triploid | NA | - | - | - | - | - | - | - | A | 115 |
| 5-Dx* | 2.57 | ADR | L | NO | Triploid | NA | - | - | - | - | - | - | - | A | 210 |
| 6-Dx | 1.97 | ABD+T | L + SKN + PS | NO | Triploid | NA | - | - | - | - | LOH | - |  | A | 220 |
| 7-Dx* | 4.87 | ADR | L+ SKN + PL | NO | Triploid | NA | - | - | - | - | LOH | - | - | A | 210 |
| 8-rel* | 0.49 | ADR | L + BM | NO | Triploid | NA | - | - | - | - | - | - |  | A | 230 |
| 9-Dx* | 0.10 | ADR | L | *** | Triploid | NA |  | - | - | - |  | - | - | A | 70 |
| 10-Dx* | 3.13 | CHEST | L | NO | Triploid | NA |  |  |  |  |  |  |  | A | 130 |
| **MYCN NA** |  |  |  |  |  |  |  |  |  |  |  |  |  |  |  |
| 11-Dx* | 9.93 | ADR | B + BM | N5 | Diploid | NA | LOH | LOH | LOH | - | - | - | - | A | 225 |
| 12-Dx* | 9.93 | ADR | B + BM + L | N6 | Tetraploid | NA | LOH | LOH | - | - | - | - | + | A | 167 |
| 13-rel | 6.88 | ADR | B + BM | CCG |  | NA | LOH | LOH | - | - | - | - |  | D | 17 |
| 14-rel | 1.68 | ADR | B + BM + L + SKN | N6 | Diploid | NA | LOH | LOH | - | - | - | - |  | D | 63 |
| 15-Dx* | 12.00 | RP | B + BM | COG | Diploid | NA | LOH | LOH | - | - | - | - | + | D | 93 |
| 16-Dx* | 15.13 | RP | B + BM + L | N5 | Triploid | NA | LOH | - |  | LOH | - | - | - | A | 227 |
| 17-rel | 3.62 | ADR | BM + L + SKN | CCG | Diploid | NA | LOH | - | LOH | LOH | - | LOH |  | D | 121 |
| 18-Dx | 4.51 | ADR | B + BM + L | N5 | Diploid | NA | LOH | - | LOH | - | LOH | LOH |  | A | 153 |
| 19-Dx* | 11.74 | CHEST | B + BM | N7 | Diploid | NA | LOH | - | - | LOH | LOH | LOH | + | A | 147 |
| 20-Dx* | 11.35 | ADR | B | N6 | Diploid | NA | LOH | - | - | LOH | LOH | LOH | - | A | 156 |
| 21-Dx* | 6.91 | ADR | B + BM | N6 | Tetraploid | NA | - | - | - | - | LOH | LOH | - | A | 192 |
| 22-Dx* | 7.89 | ADR | B + BM | N5 | Tetraploid | NA | - | - | - | - | LOH | - | + | A | 91 |
| 23-Dx* | 16.71 | CHEST | BM | N0 | Triploid | NA | - | - | - | - | - | - | - | D | 66 |
| 24-Dx* | 0.59 | ABD | L + Br + Leptomening | CCG |  | NA | - | - |  | - | - |  | - | D | 0.1 |
| **MYCN A** |  |  |  |  |  |  |  |  |  |  |  |  |  |  |  |
| 26-Dx | 9.28 | ADR | B + BM + L | N7 | Diploid | A | LOH | LOH | LOH | LOH | - | - | + | D | 8 |
| 27-Dx | 8.91 | RP | B | N7 | Diploid | A | LOH | LOH | LOH | LOH | - | - |  | D | 16 |
| 28-Dx* | 12.96 | ADR | B + BM | N7 |  | A | LOH | LOH |  | LOH | - | - | + | A | 123 |
| 29-Dx* | 12.89 | ADR | B + BM | N7 | Diploid | A | LOH | LOH |  | LOH | - | - | + | D | 24 |
| 25-Dx* | 6.32 | ADR | B + BM | N7 | Diploid | A | LOH | LOH | - | - | - | - | - | D | 2 |
| 30-Dx* | 15.07 | CHEST | B + BM | N7 | Diploid | A | LOH | LOH | - | - | - | - | - | D | 11 |
| 31-Dx* | 5.46 | ADR | B | N7 | Diploid | A | LOH | - | LOH | - | - | LOH | - | A | 99 |
| 32-Dx* | 5.69 | ABD+T | B + BM | N6 | Diploid | A | - | - | - | - | - | - |  | D | 3 |
| 33-Dx* | 7.63 | ADR | T | POG | Diploid | A |  |  |  |  |  |  | + | D | 9 |
| 34-Dx* | 4.05 | ADR | B + BM + L + Br | N8 |  | A |  |  |  |  |  |  |  | A | 65 |
| 35-Dx* | 12.20 | ADR | B + BM | N/ | Diploid | A |  |  |  |  |  |  |  | A | 126 |
