# Supplementary figures and images for: Specific gene expression profiles and chromosomal abnormalities are associated with infant disseminated neuroblastoma
Source: BMC Cancer. 2009 Feb 3;9:44. doi: 10.1186/1471-2407-9-44 (PMC2642835; doi:10.1186/1471-2407-9-44)

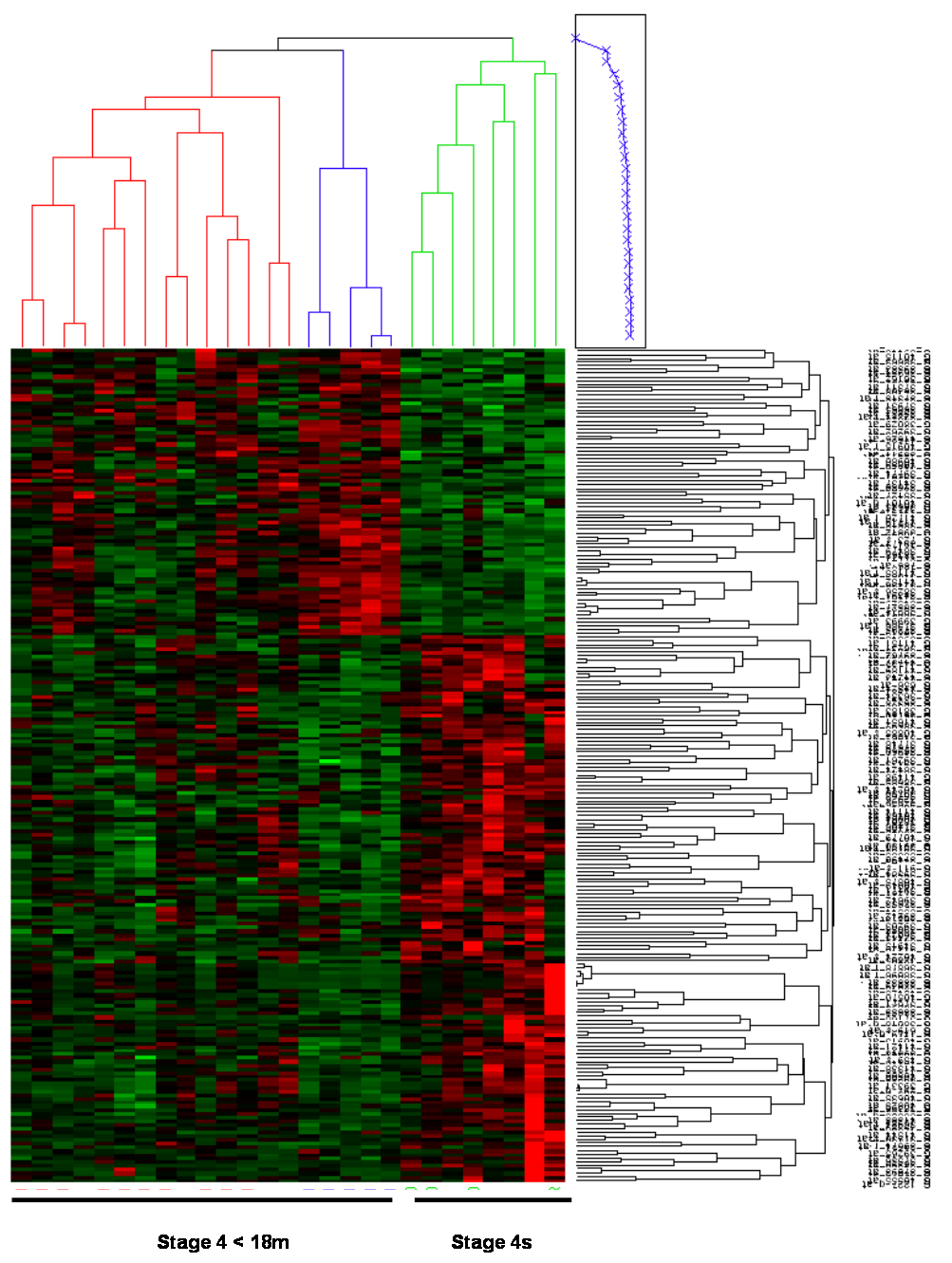

Supplement: Additional file 4 — Hierarchical clustering and heatmap representation of gene expression profiles. Comparison analysis between stage 4s and stage 4 NB tumours < 18 months: 220 differentially expressed genes. [file 1471-2407-9-44-S4.tiff]

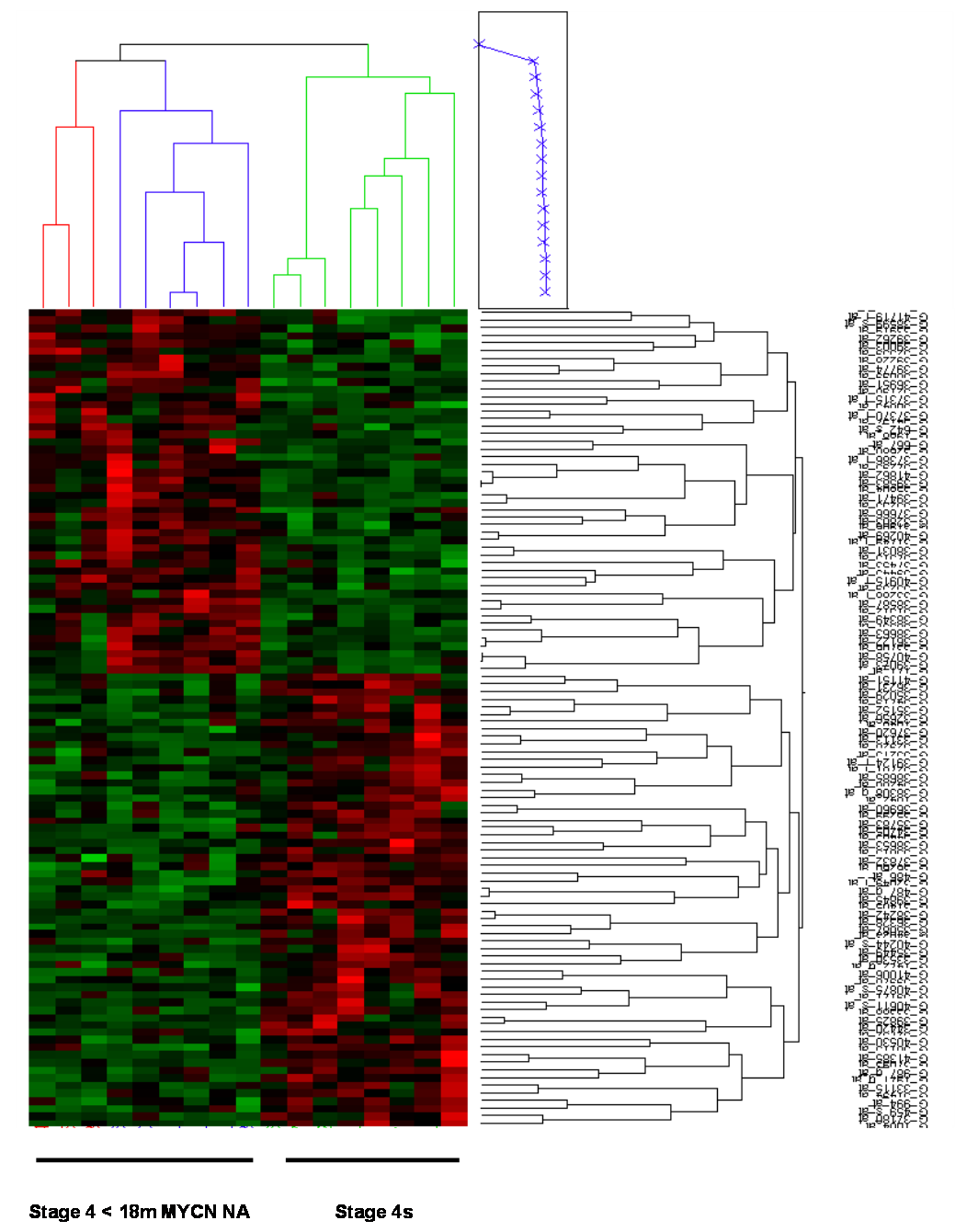

Supplement: Additional file 5 — Hierarchical clustering and heatmap representation of gene expression profiles. Comparison analysis between stage 4s and stage 4 NB tumours < 18 months without MYCN amplification: 107 differentially expressed genes. [file 1471-2407-9-44-S5.tiff]
