# Supplementary material for: Specific gene expression profiles and chromosomal abnormalities are associated with infant disseminated neuroblastoma
Source: BMC Cancer. 2009 Feb 3;9:44. doi: 10.1186/1471-2407-9-44 (PMC2642835; doi:10.1186/1471-2407-9-44)
Supplement: Additional file 6 — Intersectional Venn diagram analysis of differentially expressed gene probe sets. Intersectional Venn diagram analysis of differentially expressed gene probe set ID reported in Additional file 3. The number of probe sets common between stage 4s versus stage 4 < 12m and stage 4s versus stage 4 < 18m was higher than with stage 4s versus stage 4 <18m MYCN NA; 124/233 (53%) and 124/224 (55%) versus 51/107 (47%) and 48/107 (45%) probe sets, respectively. Thirty-eight probe sets were found common among all three gene lists. [file 1471-2407-9-44-S6.doc]

**Additional file 6**

**Intersectional Venn diagram analysis of expressed gene probe set ID reported in Additional file 3.**


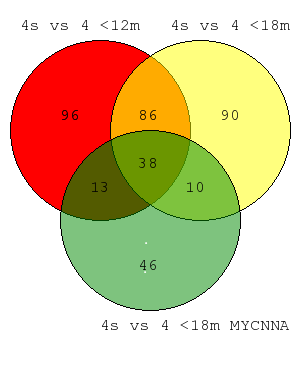


Found in probe set list stage 4s vs. stage 4 <12m only

==========

1211_s_at

1243_at

1255_g_at

1450_g_at

1496_at

1645_at

1676_s_at

1838_g_at

1980_s_at

275_at

31400_at

315_at

31609_s_at

31693_f_at

31873_at

31892_at

32051_at

32193_at

32728_at

32798_at

32820_at

32844_at

32919_at

33198_at

33289_f_at

33345_at

33392_at

33415_at

33473_at

33514_at

34144_at

34160_at

34171_at

34295_at

34336_at

34346_at

34369_at

34413_at

34685_at

34726_at

34803_at

34835_at

34846_at

34861_at

35278_at

35279_at

35351_at

36044_at

36166_at

36279_at

36687_at

36717_at

36812_at

36847_r_at

37298_at

37362_at

37365_at

37389_at

37726_at

37808_at

37984_s_at

38150_at

38307_at

38342_at

38435_at

38461_at

39102_at

39107_at

39157_at

39196_i_at

39396_at

39653_at

39732_at

39765_at

39865_at

40139_at

40140_at

40175_at

40179_at

40276_at

40470_at

40509_at

40524_at

40628_at

40839_at

40861_at

41055_at

41160_at

41188_at

41396_at

41485_at

41632_at

41872_at

829_s_at

831_at

876_at

Found in the probe set list stage 4s vs. stage 4 <18m only

==========

1227_g_at

1248_at

1478_at

1479_g_at

1515_at

1641_s_at

2025_s_at

2028_s_at

2064_g_at

31320_at

31802_at

31918_at

31956_f_at

32181_at

32217_at

32274_r_at

32576_at

32743_at

32805_at

33340_at

33819_at

34085_at

34150_at

34226_at

34267_r_at

34391_at

34829_at

34862_at

34877_at

34915_at

35016_at

35127_at

35329_at

35390_at

35733_at

36096_at

36312_at

36354_at

36773_f_at

36780_at

36785_at

36878_f_at

36894_at

37039_at

37168_at

37185_at

37287_at

37406_at

37530_s_at

37556_at

37671_at

37731_at

37748_at

37931_at

38018_g_at

38029_at

38095_i_at

38096_f_at

38396_at

38692_at

38704_at

38833_at

39058_at

39203_at

39265_at

39288_at

39331_at

39370_at

39750_at

40101_g_at

40224_s_at

40423_at

40555_at

40570_at

40635_at

40779_at

40825_at

40859_at

40913_at

40980_at

41114_at

41185_f_at

41243_at

41338_at

41544_at

41626_at

41651_at

41720_r_at

41737_at

656_at

Found in the probe set list stage 4s vs. stage 4 <18m MYCN NA only

==========

1472_g_at

1971_g_at

1988_at

31312_at

31405_at

31749_f_at

31906_at

32732_at

32803_at

33113_at

33115_at

33259_at

33530_at

34483_at

35029_at

35299_at

35804_at

36260_at

36397_at

37180_at

37313_at

37370_i_at

37453_at

37620_at

37666_at

37832_at

38031_at

38113_at

38242_at

38349_at

38587_at

39003_at

39073_at

39124_r_at

39228_at

39825_at

39978_at

40269_at

40530_at

40611_s_at

41006_at

41862_at

487_g_at

642_s_at

667_at

994_at

Found in the probe set lists stage 4s vs. stage 4 <12m and stage 4s vs. stage 4 MYCN NA <18m ==========

1004_at

1842_at

32049_f_at

32359_at

33613_at

33706_at

33919_at

34705_at

34719_at

38599_s_at

40758_at

486_at

987_g_at

Found in the probe set lists stage 4s vs. stage 4 <12m and stage 4s vs. stage 4 <18m

==========

1252_at

1979_s_at

296_at

297_g_at

31882_at

32002_at

32028_at

32076_at

32093_at

32209_at

32253_at

32254_at

32415_at

32436_at

32487_s_at

32554_s_at

32727_at

32780_at

32852_at

33133_at

33207_at

33235_at

33341_at

33365_at

33458_r_at

33881_at

34240_s_at

34731_at

35087_at

35281_at

35308_at

35336_at

35620_at

35695_at

35762_at

35814_at

36024_at

36144_at

36162_at

362_at

36844_at

36865_at

36924_r_at

37060_at

37286_at

37311_at

37490_at

37653_at

37679_at

37741_at

38183_at

38256_s_at

38368_at

38424_at

38479_at

38527_at

38618_at

38635_at

38911_at

39089_at

39150_at

39242_at

39261_at

39348_at

39584_at

39642_at

39674_r_at

39762_at

39812_at

39993_at

40049_at

40064_at

40115_at

40446_at

40468_at

40500_at

40885_s_at

41152_f_at

41189_at

41421_at

41498_at

41524_at

619_s_at

723_s_at

786_at

911_s_at

Found in the probe set lists stage 4s vs. stage 4 <18m and stage 4s vs. stage 4 MYCN NA <18m ==========

171_at

32926_at

34137_at

34420_at

35449_at

39262_at

39471_at

39774_at

40875_s_at

40915_r_at

Found in all three probe set lists

==========

35783_at

39171_at

34200_at

38685_at

38308_g_at

36960_at

34023_at

1846_at

33388_at

39845_at

459_s_at

31494_at

32781_f_at

35152_at

32656_at

37093_at

35213_at

41151_at

36328_at

38653_at

41385_at

40244_s_at

36231_at

39445_at

39583_at

32680_at

37295_at

35255_at

32190_at

41719_i_at

36645_at

36951_at

38663_at

36122_at

37315_f_at

33288_i_at

37386_i_at

36699_at
